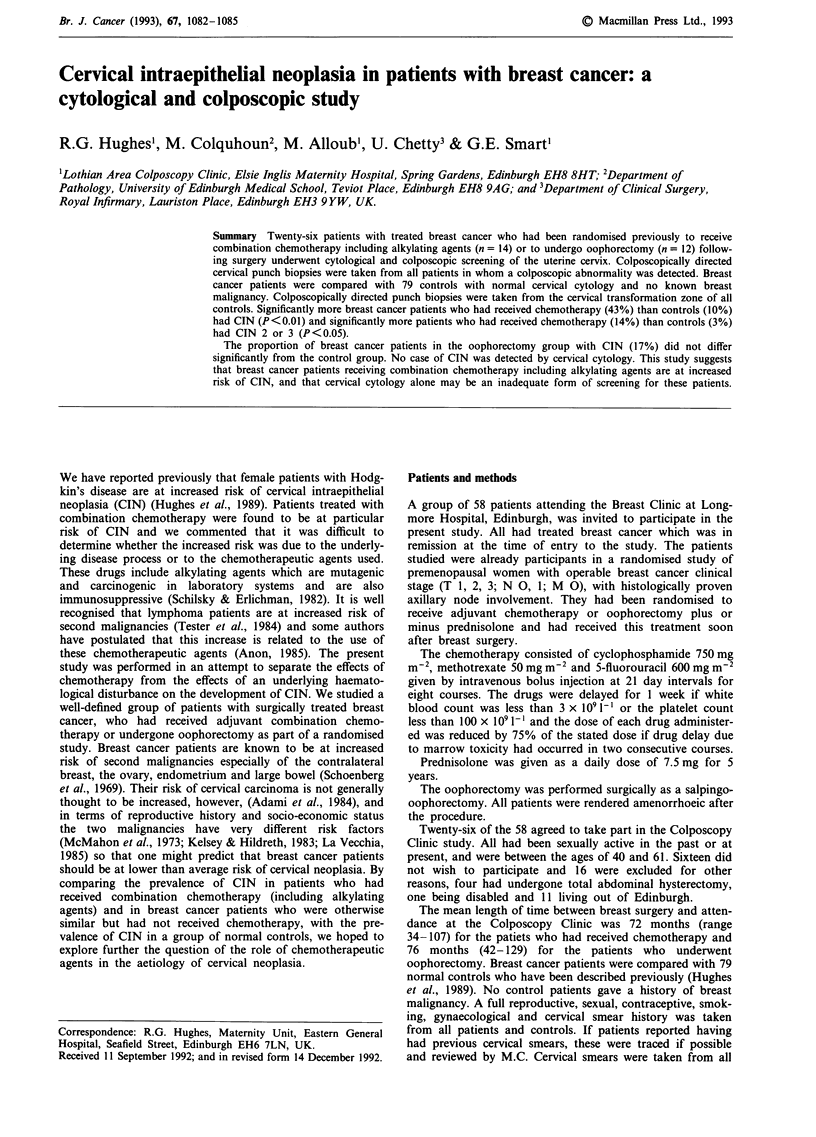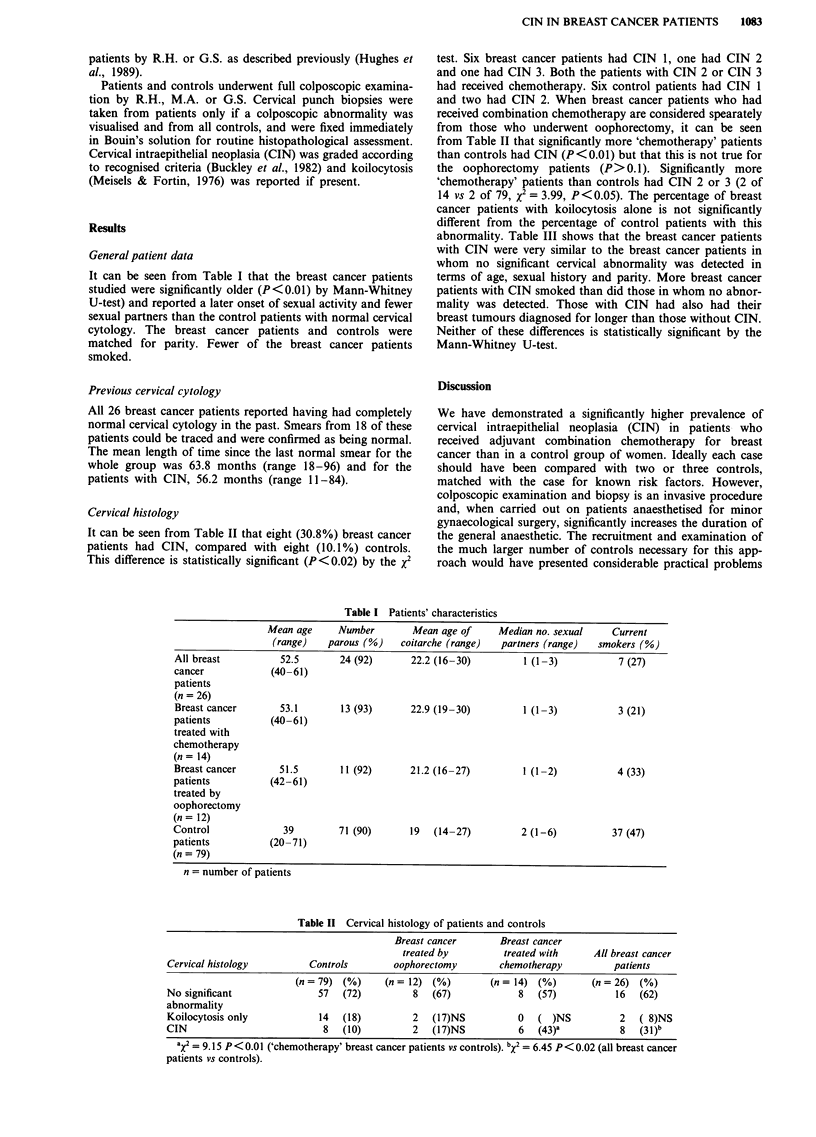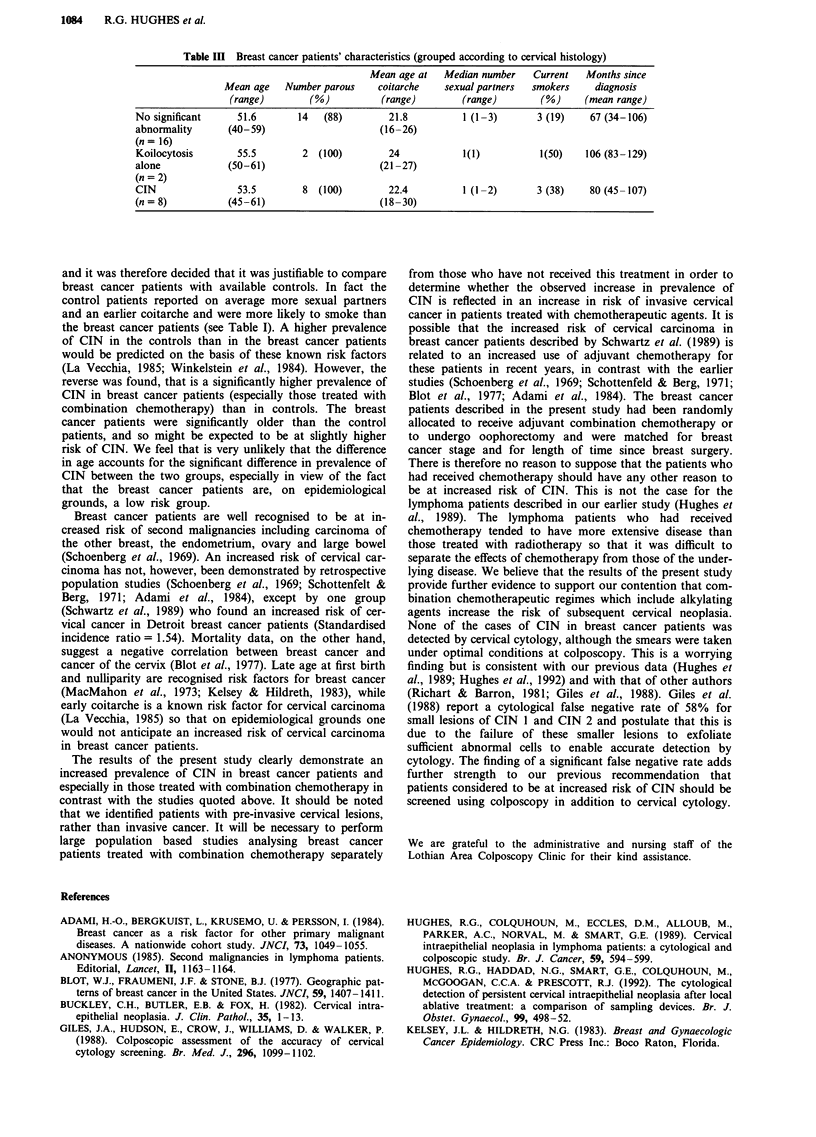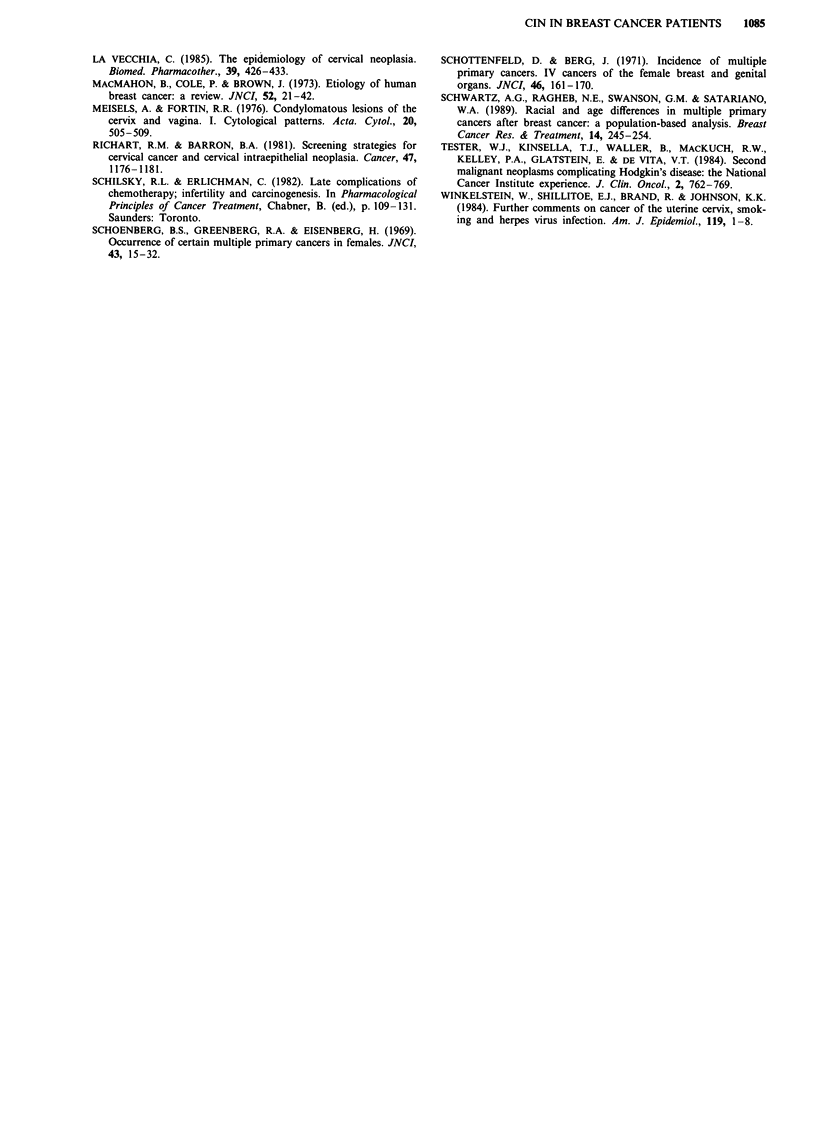# Cervical intraepithelial neoplasia in patients with breast cancer: a cytological and colposcopic study.

**DOI:** 10.1038/bjc.1993.198

**Published:** 1993-05

**Authors:** R. G. Hughes, M. Colquhoun, M. Alloub, U. Chetty, G. E. Smart

**Affiliations:** Lothian Area Colposcopy Clinic, Elsie Inglis Maternity Hospital, Spring Gardens, Edinburgh, UK.

## Abstract

Twenty-six patients with treated breast cancer who had been randomised previously to receive combination chemotherapy including alkylating agents (n = 14) or to undergo oophorectomy (n = 12) following surgery underwent cytological and colposcopic screening of the uterine cervix. Colposcopically directed cervical punch biopsies were taken from all patients in whom a colposcopic abnormality was detected. Breast cancer patients were compared with 79 controls with normal cervical cytology and no known breast malignancy. Colposcopically directed punch biopsies were taken from the cervical transformation zone of all controls. Significantly more breast cancer patients who had received chemotherapy (43%) than controls (10%) had CIN (P < 0.01) and significantly more patients who had received chemotherapy (14%) than controls (3%) had CIN 2 or 3 (P < 0.05). The proportion of breast cancer patients in the oophorectomy group with CIN (17%) did not differ significantly from the control group. No case of CIN was detected by cervical cytology. This study suggests that breast cancer patients receiving combination chemotherapy including alkylating agents are at increased risk of CIN, and that cervical cytology alone may be an inadequate form of screening for these patients.


### Cervical intraepithelial neoplasia in patients with breast cancer: a cytological and colposcopic study

R.G. Hughes[1], M. Colquhoun[2], M. Alloub[1], U. Chetty[3] & G.E. Smart[1]

[1]*Lothian Area Colposcopy Clinic, Elsie Inglis Maternity Hospital, Spring Gardens, Edinburgh EH8 8HT;* [2]*Department of Pathology, University of Edinburgh Medical School, Teviot Place, Edinburgh EH8 9AG; and* [3]*Department of Clinical Surgery, Royal Infirmary, Lauriston Place, Edinburgh EH3 9YW, UK.*

**Summary** Twenty-six patients with treated breast cancer who had been randomised previously to receive combination chemotherapy including alkylating agents ($n = 14$) or to undergo oophorectomy ($n = 12$) following surgery underwent cytological and colposcopic screening of the uterine cervix. Colposcopically directed cervical punch biopsies were taken from all patients in whom a colposcopic abnormality was detected. Breast cancer patients were compared with 79 controls with normal cervical cytology and no known breast malignancy. Colposcopically directed punch biopsies were taken from the cervical transformation zone of all controls. Significantly more breast cancer patients who had received chemotherapy (43%) than controls (10%) had CIN ($P < 0.01$) and significantly more patients who had received chemotherapy (14%) than controls (3%) had CIN 2 or 3 ($P < 0.05$).

The proportion of breast cancer patients in the oophorectomy group with CIN (17%) did not differ significantly from the control group. No case of CIN was detected by cervical cytology. This study suggests that breast cancer patients receiving combination chemotherapy including alkylating agents are at increased risk of CIN, and that cervical cytology alone may be an inadequate form of screening for these patients.

We have reported previously that female patients with Hodgkin's disease are at increased risk of cervical intraepithelial neoplasia (CIN) (Hughes *et al.*, 1989). Patients treated with combination chemotherapy were found to be at particular risk of CIN and we commented that it was difficult to determine whether the increased risk was due to the underlying disease process or to the chemotherapeutic agents used. These drugs include alkylating agents which are mutagenic and carcinogenic in laboratory systems and are also immunosuppressive (Schilsky & Erlichman, 1982). It is well recognised that lymphoma patients are at increased risk of second malignancies (Tester *et al.*, 1984) and some authors have postulated that this increase is related to the use of these chemotherapeutic agents (Anon, 1985). The present study was performed in an attempt to separate the effects of chemotherapy from the effects of an underlying haematological disturbance on the development of CIN. We studied a well-defined group of patients with surgically treated breast cancer, who had received adjuvant combination chemotherapy or undergone oophorectomy as part of a randomised study. Breast cancer patients are known to be at increased risk of second malignancies especially of the contralateral breast, the ovary, endometrium and large bowel (Schoenberg *et al.*, 1969). Their risk of cervical carcinoma is not generally thought to be increased, however, (Adami *et al.*, 1984), and in terms of reproductive history and socio-economic status the two malignancies have very different risk factors (McMahon *et al.*, 1973; Kelsey & Hildreth, 1983; La Vecchia, 1985) so that one might predict that breast cancer patients should be at lower than average risk of cervical neoplasia. By comparing the prevalence of CIN in patients who had received combination chemotherapy (including alkylating agents) and in breast cancer patients who were otherwise similar but had not received chemotherapy, with the prevalence of CIN in a group of normal controls, we hoped to explore further the question of the role of chemotherapeutic agents in the aetiology of cervical neoplasia.

Correspondence: R.G. Hughes, Maternity Unit, Eastern General Hospital, Seafield Street, Edinburgh EH6 7LN, UK.
Received 11 September 1992; and in revised form 14 December 1992.

### Patients and methods

A group of 58 patients attending the Breast Clinic at Longmore Hospital, Edinburgh, was invited to participate in the present study. All had treated breast cancer which was in remission at the time of entry to the study. The patients studied were already participants in a randomised study of premenopausal women with operable breast cancer clinical stage (T 1, 2, 3; N O, 1; M O), with histologically proven axillary node involvement. They had been randomised to receive adjuvant chemotherapy or oophorectomy plus or minus prednisolone and had received this treatment soon after breast surgery.

The chemotherapy consisted of cyclophosphamide 750 mg $m^{-2}$, methotrexate 50 mg $m^{-2}$ and 5-fluorouracil 600 mg $m^{-2}$ given by intravenous bolus injection at 21 day intervals for eight courses. The drugs were delayed for 1 week if white blood count was less than $3 \times 10^9 l^{-1}$ or the platelet count less than $100 \times 10^9 l^{-1}$ and the dose of each drug administered was reduced by 75% of the stated dose if drug delay due to marrow toxicity had occurred in two consecutive courses.

Prednisolone was given as a daily dose of 7.5 mg for 5 years.

The oophorectomy was performed surgically as a salpingo-oophorectomy. All patients were rendered amenorrhoeic after the procedure.

Twenty-six of the 58 agreed to take part in the Colposcopy Clinic study. All had been sexually active in the past or at present, and were between the ages of 40 and 61. Sixteen did not wish to participate and 16 were excluded for other reasons, four had undergone total abdominal hysterectomy, one being disabled and 11 living out of Edinburgh.

The mean length of time between breast surgery and attendance at the Colposcopy Clinic was 72 months (range 34-107) for the patiets who had received chemotherapy and 76 months (42-129) for the patients who underwent oophorectomy. Breast cancer patients were compared with 79 normal controls who have been described previously (Hughes *et al.*, 1989). No control patients gave a history of breast malignancy. A full reproductive, sexual, contraceptive, smoking, gynaecological and cervical smear history was taken from all patients and controls. If patients reported having had previous cervical smears, these were traced if possible and reviewed by M.C. Cervical smears were taken from all patients by R.H. or G.S. as described previously (Hughes *et al.*, 1989).

Patients and controls underwent full colposcopic examination by R.H., M.A. or G.S. Cervical punch biopsies were taken from patients only if a colposcopic abnormality was visualised and from all controls, and were fixed immediately in Bouin's solution for routine histopathological assessment. Cervical intraepithelial neoplasia (CIN) was graded according to recognised criteria (Buckley *et al.*, 1982) and koilocytosis (Meisels & Fortin, 1976) was reported if present.

### Results

### *General patient data*

It can be seen from Table I that the breast cancer patients studied were significantly older ($P<0.01$) by Mann-Whitney U-test) and reported a later onset of sexual activity and fewer sexual partners than the control patients with normal cervical cytology. The breast cancer patients and controls were matched for parity. Fewer of the breast cancer patients smoked.

### *Previous cervical cytology*

All 26 breast cancer patients reported having had completely normal cervical cytology in the past. Smears from 18 of these patients could be traced and were confirmed as being normal. The mean length of time since the last normal smear for the whole group was 63.8 months (range 18–96) and for the patients with CIN, 56.2 months (range 11–84).

### *Cervical histology*

It can be seen from Table II that eight (30.8%) breast cancer patients had CIN, compared with eight (10.1%) controls. This difference is statistically significant ($P<0.02$) by the $\chi^2$ test. Six breast cancer patients had CIN 1, one had CIN 2 and one had CIN 3. Both the patients with CIN 2 or CIN 3 had received chemotherapy. Six control patients had CIN 1 and two had CIN 2. When breast cancer patients who had received combination chemotherapy are considered spearately from those who underwent oophorectomy, it can be seen from Table II that significantly more 'chemotherapy' patients than controls had CIN ($P<0.01$) but that this is not true for the oophorectomy patients ($P>0.1$). Significantly more 'chemotherapy' patients than controls had CIN 2 or 3 (2 of 14 *vs* 2 of 79, $\chi^2 = 3.99$, $P<0.05$). The percentage of breast cancer patients with koilocytosis alone is not significantly different from the percentage of control patients with this abnormality. Table III shows that the breast cancer patients with CIN were very similar to the breast cancer patients in whom no significant cervical abnormality was detected in terms of age, sexual history and parity. More breast cancer patients with CIN smoked than did those in whom no abnormality was detected. Those with CIN had also had their breast tumours diagnosed for longer than those without CIN. Neither of these differences is statistically significant by the Mann-Whitney U-test.

### Discussion

We have demonstrated a significantly higher prevalence of cervical intraepithelial neoplasia (CIN) in patients who received adjuvant combination chemotherapy for breast cancer than in a control group of women. Ideally each case should have been compared with two or three controls, matched with the case for known risk factors. However, colposcopic examination and biopsy is an invasive procedure and, when carried out on patients anaesthetised for minor gynaecological surgery, significantly increases the duration of the general anaesthetic. The recruitment and examination of the much larger number of controls necessary for this approach would have presented considerable practical problems

**Table I** Patients' characteristics

| | *Mean age (range)* | *Number parous (%)* | *Mean age of coitarche (range)* | *Median no. sexual partners (range)* | *Current smokers (%)* |
|---|---|---|---|---|---|
| All breast cancer patients ($n = 26$) | 52.5 (40–61) | 24 (92) | 22.2 (16–30) | 1 (1–3) | 7 (27) |
| Breast cancer patients treated with chemotherapy ($n = 14$) | 53.1 (40–61) | 13 (93) | 22.9 (19–30) | 1 (1–3) | 3 (21) |
| Breast cancer patients treated by oophorectomy ($n = 12$) | 51.5 (42–61) | 11 (92) | 21.2 (16–27) | 1 (1–2) | 4 (33) |
| Control patients ($n = 79$) | 39 (20–71) | 71 (90) | 19 (14–27) | 2 (1–6) | 37 (47) |

$n$ = number of patients

**Table II** Cervical histology of patients and controls

| *Cervical histology* | *Controls* | | *Breast cancer treated by oophorectomy* | | *Breast cancer treated with chemotherapy* | | *All breast cancer patients* | |
|---|---|---|---|---|---|---|---|---|
| | ($n = 79$) | (%) | ($n = 12$) | (%) | ($n = 14$) | (%) | ($n = 26$) | (%) |
| No significant abnormality | 57 | (72) | 8 | (67) | 8 | (57) | 16 | (62) |
| Koilocytosis only | 14 | (18) | 2 | (17)NS | 0 | ( )NS | 2 | ( 8)NS |
| CIN | 8 | (10) | 2 | (17)NS | 6 | (43)[a] | 8 | (31)[b] |

[a]$\chi^2 = 9.15$ $P<0.01$ ('chemotherapy' breast cancer patients *vs* controls). [b]$\chi^2 = 6.45$ $P<0.02$ (all breast cancer patients *vs* controls).

**Table III** Breast cancer patients' characteristics (grouped according to cervical histology)

| | *Mean age (range)* | *Number parous (%)* | *Mean age at coitarche (range)* | *Median number sexual partners (range)* | *Current smokers (%)* | *Months since diagnosis (mean range)* |
|---|---|---|---|---|---|---|
| No significant abnormality ($n = 16$) | 51.6 (40–59) | 14 (88) | 21.8 (16–26) | 1 (1–3) | 3 (19) | 67 (34–106) |
| Koilocytosis alone ($n = 2$) | 55.5 (50–61) | 2 (100) | 24 (21–27) | 1(1) | 1(50) | 106 (83–129) |
| CIN ($n = 8$) | 53.5 (45–61) | 8 (100) | 22.4 (18–30) | 1 (1–2) | 3 (38) | 80 (45–107) |

and it was therefore decided that it was justifiable to compare breast cancer patients with available controls. In fact the control patients reported on average more sexual partners and an earlier coitarche and were more likely to smoke than the breast cancer patients (see Table I). A higher prevalence of CIN in the controls than in the breast cancer patients would be predicted on the basis of these known risk factors (La Vecchia, 1985; Winkelstein *et al.*, 1984). However, the reverse was found, that is a significantly higher prevalence of CIN in breast cancer patients (especially those treated with combination chemotherapy) than in controls. The breast cancer patients were significantly older than the control patients, and so might be expected to be at slightly higher risk of CIN. We feel that is very unlikely that the difference in age accounts for the significant difference in prevalence of CIN between the two groups, especially in view of the fact that the breast cancer patients are, on epidemiological grounds, a low risk group.

Breast cancer patients are well recognised to be at increased risk of second malignancies including carcinoma of the other breast, the endometrium, ovary and large bowel (Schoenberg *et al.*, 1969). An increased risk of cervical carcinoma has not, however, been demonstrated by retrospective population studies (Schoenberg *et al.*, 1969; Schottenfelt & Berg, 1971; Adami *et al.*, 1984), except by one group (Schwartz *et al.*, 1989) who found an increased risk of cervical cancer in Detroit breast cancer patients (Standardised incidence ratio = 1.54). Mortality data, on the other hand, suggest a negative correlation between breast cancer and cancer of the cervix (Blot *et al.*, 1977). Late age at first birth and nulliparity are recognised risk factors for breast cancer (MacMahon *et al.*, 1973; Kelsey & Hildreth, 1983), while early coitarche is a known risk factor for cervical carcinoma (La Vecchia, 1985) so that on epidemiological grounds one would not anticipate an increased risk of cervical carcinoma in breast cancer patients.

The results of the present study clearly demonstrate an increased prevalence of CIN in breast cancer patients and especially in those treated with combination chemotherapy in contrast with the studies quoted above. It should be noted that we identified patients with pre-invasive cervical lesions, rather than invasive cancer. It will be necessary to perform large population based studies analysing breast cancer patients treated with combination chemotherapy separately from those who have not received this treatment in order to determine whether the observed increase in prevalence of CIN is reflected in an increase in risk of invasive cervical cancer in patients treated with chemotherapeutic agents. It is possible that the increased risk of cervical carcinoma in breast cancer patients described by Schwartz *et al.* (1989) is related to an increased use of adjuvant chemotherapy for these patients in recent years, in contrast with the earlier studies (Schoenberg *et al.*, 1969; Schottenfeld & Berg, 1971; Blot *et al.*, 1977; Adami *et al.*, 1984). The breast cancer patients described in the present study had been randomly allocated to receive adjuvant combination chemotherapy or to undergo oophorectomy and were matched for breast cancer stage and for length of time since breast surgery. There is therefore no reason to suppose that the patients who had received chemotherapy should have any other reason to be at increased risk of CIN. This is not the case for the lymphoma patients described in our earlier study (Hughes *et al.*, 1989). The lymphoma patients who had received chemotherapy tended to have more extensive disease than those treated with radiotherapy so that it was difficult to separate the effects of chemotherapy from those of the underlying disease. We believe that the results of the present study provide further evidence to support our contention that combination chemotherapeutic regimes which include alkylating agents increase the risk of subsequent cervical neoplasia. None of the cases of CIN in breast cancer patients was detected by cervical cytology, although the smears were taken under optimal conditions at colposcopy. This is a worrying finding but is consistent with our previous data (Hughes *et al.*, 1989; Hughes *et al.*, 1992) and with that of other authors (Richart & Barron, 1981; Giles *et al.*, 1988). Giles *et al.* (1988) report a cytological false negative rate of 58% for small lesions of CIN 1 and CIN 2 and postulate that this is due to the failure of these smaller lesions to exfoliate sufficient abnormal cells to enable accurate detection by cytology. The finding of a significant false negative rate adds further strength to our previous recommendation that patients considered to be at increased risk of CIN should be screened using colposcopy in addition to cervical cytology.

We are grateful to the administrative and nursing staff of the Lothian Area Colposcopy Clinic for their kind assistance.

### References

ADAMI, H.-O., BERGKUIST, L., KRUSEMO, U. & PERSSON, I. (1984). Breast cancer as a risk factor for other primary malignant diseases. A nationwide cohort study. *JNCI*, **73**, 1049–1055.

ANONYMOUS (1985). Second malignancies in lymphoma patients. Editorial, *Lancet*, **II**, 1163–1164.

BLOT, W.J., FRAUMENI, J.F. & STONE, B.J. (1977). Geographic patterns of breast cancer in the United States. *JNCI*, **59**, 1407–1411.

BUCKLEY, C.H., BUTLER, E.B. & FOX, H. (1982). Cervical intraepithelial neoplasia. *J. Clin. Pathol.*, **35**, 1–13.

GILES, J.A., HUDSON, E., CROW, J., WILLIAMS, D. & WALKER, P. (1988). Colposcopic assessment of the accuracy of cervical cytology screening. *Br. Med. J.*, **296**, 1099–1102.

HUGHES, R.G., COLQUHOUN, M., ECCLES, D.M., ALLOUB, M., PARKER, A.C., NORVAL, M. & SMART, G.E. (1989). Cervical intraepithelial neoplasia in lymphoma patients: a cytological and colposcopic study. *Br. J. Cancer*, **59**, 594–599.

HUGHES, R.G., HADDAD, N.G., SMART, G.E., COLQUHOUN, M., MCGOOGAN, C.C.A. & PRESCOTT, R.J. (1992). The cytological detection of persistent cervical intraepithelial neoplasia after local ablative treatment: a comparison of sampling devices. *Br. J. Obstet. Gynaecol.*, **99**, 498–52.

KELSEY, J.L. & HILDRETH, N.G. (1983). *Breast and Gynaecologic Cancer Epidemiology*. CRC Press Inc.: Boco Raton, Florida.

LA VECCHIA, C. (1985). The epidemiology of cervical neoplasia. *Biomed. Pharmacother.*, **39**, 426–433.

MACMAHON, B., COLE, P. & BROWN, J. (1973). Etiology of human breast cancer: a review. *JNCI*, **52**, 21–42.

MEISELS, A. & FORTIN, R.R. (1976). Condylomatous lesions of the cervix and vagina. I. Cytological patterns. *Acta. Cytol.*, **20**, 505–509.

RICHART, R.M. & BARRON, B.A. (1981). Screening strategies for cervical cancer and cervical intraepithelial neoplasia. *Cancer*, **47**, 1176–1181.

SCHILSKY, R.L. & ERLICHMAN, C. (1982). Late complications of chemotherapy; infertility and carcinogenesis. In *Pharmacological Principles of Cancer Treatment*, Chabner, B. (ed.), p. 109–131. Saunders: Toronto.

SCHOENBERG, B.S., GREENBERG, R.A. & EISENBERG, H. (1969). Occurrence of certain multiple primary cancers in females. *JNCI*, **43**, 15–32.

SCHOTTENFELD, D. & BERG, J. (1971). Incidence of multiple primary cancers. IV cancers of the female breast and genital organs. *JNCI*, **46**, 161–170.

SCHWARTZ, A.G., RAGHEB, N.E., SWANSON, G.M. & SATARIANO, W.A. (1989). Racial and age differences in multiple primary cancers after breast cancer: a population-based analysis. *Breast Cancer Res. & Treatment*, **14**, 245–254.

TESTER, W.J., KINSELLA, T.J., WALLER, B., MACKUCH, R.W., KELLEY, P.A., GLATSTEIN, E. & DE VITA, V.T. (1984). Second malignant neoplasms complicating Hodgkin's disease: the National Cancer Institute experience. *J. Clin. Oncol.*, **2**, 762–769.

WINKELSTEIN, W., SHILLITOE, E.J., BRAND, R. & JOHNSON, K.K. (1984). Further comments on cancer of the uterine cervix, smoking and herpes virus infection. *Am. J. Epidemiol.*, **119**, 1–8.